# *Escherichia coli* Isolated from Urinary Tract Infections of Lebanese Patients between 2005 and 2012: Epidemiology and Profiles of Resistance

**DOI:** 10.3389/fmed.2015.00026

**Published:** 2015-04-28

**Authors:** Ziad Daoud, Elie Salem Sokhn, Khalil Masri, Katia Cheaito, Nathaline Haidar-Ahmad, Ghassan M. Matar, Shira Doron

**Affiliations:** ^1^Faculty of Medicine and Medical Sciences, University of Balamand, Tripoli, Lebanon; ^2^“Centre Hospitalier du Nord” Hospital, Zgharta, Lebanon; ^3^Faculty of Medicine, American University of Beirut, Beirut, Lebanon; ^4^Division of Geographic and Infectious Diseases, Tufts Medical Center, Boston, MA, USA

**Keywords:** ESBL, carbapenemases, *E. coli*, urinary infections, bacterial resistance

## Abstract

The early treatment of urinary tract infections (UTIs) is directly related to decrease in morbidity, which makes the empirical treatment of great importance. Recently, beta lactamases of several types have emerged as significant mechanisms of resistance in Gram-negative bacilli, especially *Escherichia coli*. Our aim was to study the urinary *E. coli* isolated from Lebanese patients and to characterize their mechanisms of resistance. The study analyzed data between 2005 and 2012 of UTIs caused by *E. coli*. The mechanisms of resistance were characterized by phenotypic and genotypic methods and the pulsed-field gel electrophoresis (PFGE) was used to determine the different bacterial clusters. As expected, the highest incidence was observed with *E. coli* (60.53–73.98%) followed by *K. pneumoniae* (5.32–8.33%). ICU isolates were constantly associated with the lowest rates of susceptibility to extended-spectrum cephalosporins, ciprofloxacin, as well as most of the tested antibiotics. A 100% occurrence of CTX-M in extended-spectrum β-lactamase (ESBL)-producing isolates was recorded, followed by TEM, SHV, and OXA. In addition, 15.9% harbored 4 different ESBL enzymes and only 13 isolates (14.8%) harbored only one enzyme (CTX-M). Over the years, the simultaneous susceptibility of *E. coli* to ceftazidime and ciprofloxacin decreased from 62.5% in 2006 to 48.7% in 2012. PFGE results demonstrated that 10 clusters were 32 generated, denoting diversity among detected isolates. Understanding the epidemiology of resistance is 33 instrumental for the implementation of recommendations for the management of antimicrobials, infection 34 control measures, as well as active surveillance and antimicrobial stewardship.

## Introduction

The antimicrobials use and misuse have led to bacterial resistance. Urinary tract infections (UTIs) are among the most common infectious diseases encountered in the community and in the hospital; they result in high rates of morbidity and high economic costs associated with treatment (1–3). In recent decades, the extended-spectrum β-lactamases (ESBLs) of the TEM, SHV, CTX-M, and OXA type, as well as the CTX-M have emerged as significant mechanisms of resistance in Gram-negative bacilli (4). ESBLs are enzymes able to efficiently hydrolyze extended-spectrum cephalosporins and monobactams and have been associated with therapeutic failures. These enzymes have widely spread to geographic regions, and this is due, in part, to the fact that many resistance genes are often carried on self-transmissible or mobile plasmids that are capable of spreading horizontally between and within species (4, 5). In addition, carbapenem resistance, a worrisome public health threat, is being reported throughout the world.

Early treatment of UTI is directly related to decrease in morbidity, which makes the selection of empirical therapy of great importance (6). The correct choice of empirical antimicrobial requires a good understanding of the typical bacteriology involved in UTIs (4–6), local resistance patterns, as well as the specific patient’s antimicrobial and microbiologic history, all of which affect the relative likelihoods of various organisms. Appropriate selection of antimicrobial therapy is an important strategy in the prevention of the spread of resistance, since use of overly broad therapy can lead to development of resistance. *Escherichia coli*, a major component of the intestinal flora, has been described as the most frequent uropathogen involved in community as well as hospital-acquired UTI (6–9). According to a study previously performed in Beirut (10), *E. coli* was the most frequent isolate (60.64% of the total isolates) and an increase in the production of ESBL was observed between the years 2000 and 2009 (2.3–16.8%). Unfortunately, very limited data concerning UTIs is available from other regions of the country. The north of Lebanon represents an important site for the investigation of bacterial infection and resistance in view of the many socio-cultural, economic, and medical practice differences. The purpose of this study was to investigate the bacterial etiology of UTIs in one of the busiest hospitals of the north of Lebanon (Centre Hospitalier du Nord) and to examine the epidemiologic and microbiologic properties, including phenotypic and genotypic characterization of the mechanisms of resistance of *E. coli* isolated from UTIs of Lebanese patients over an 8-year period.

## Materials and Methods

### Study design

This study includes a retrospective part analyzing the data generated between January 2005 and January 2011, and a prospective part dealing with the occurrence of beta-lactamases genes in clinical isolates of *E. coli* between January 2011 and January 2013 and the related epidemiology of infection. Isolates of *E. coli* that showed intermediate or full resistance to carbapenems were stored at −80°C until they were processed for analysis. Over the 8-year-study, the population included all in and outpatients with positive urine cultures for a Gram-negative bacillus (6284) regardless of the type of UTI (complicated UTI, non-complicated UTI, acute pyelonephritis, etc.). Histological and microbiological criteria for positivity are cited below. In both the retrospective and prospective parts of the study, all urinary isolates data were collected and entered into a database using WHONET software. These data were classified based on frequency, department of origin, antimicrobial susceptibility profiles, as well as etiology. Among the UTIs caused by Gram-negative bacilli, there were 4,097 (65.2%) samples from female patients and 2,187 (34.8%) from males. Adult patients were sampled by clean catch midstream urine, and children aged <3 years were sampled using sterile urine bags. Only a single positive culture per patient was included in the analysis within the period of 3 months.

### Isolation and identification of organisms

Samples for urine culture were tested within half an hour of sampling. All samples were inoculated on Uriselect agar and MacConkey agar and incubated at 37°C for 24 h, and for 48 h in case no growth was observed after 24 h. A specimen was considered positive for UTI in the light of the number of yielded colonies (≥10^5^ cfu/mL) and the cytology of the urine through microscopic detection of bacteriuria and PMNs (≥8 leukocytes/mm^3^). However, lower colony counts associated with significant pyuria or low PMN count associated with significant colony counts was considered and analyzed in the light of the clinical picture and the patient’s immunological status. Bacterial identification was based on standard culture and biochemical characteristics of isolates. Gram-negative bacteria were identified by standard biochemical tests (11, 12).

### Antimicrobial susceptibility testing

Antimicrobial susceptibility of Enterobacteriaceae was tested by the disk diffusion method according to CLSI recommendations, using Mueller–Hinton agar (13). Antimicrobial agents tested were ampicillin, amoxicillin–clavulanic acid, aztreonam, cefoxitin, cefuroxime, cefotaxime, ceftriaxone, ceftazidime, cefepime, piperacillin, piperacillin–tazobactam, imipenem, meropenem, gentamicin, tobramycin, norfloxacin, ciprofloxacin, trimethoprim–sulfamethoxazole, and tigecycline (all disk charges were chosen as per the CLSI recommendations)

### Statistical analysis

Variables were expressed as percentages.

### Phenotypic detection of resistance

The phenotypic detection of ESBL was done by the double disk synergy test (DDST). In brief, ceftazidime (30 mg), cefepime (30 mg), aztreonem (30 mg), and cefotaxime (30 mg) disks (Oxoid) were placed 25 mm (center to center) from the amoxicillin/clavulanic acid (20/10 mg) disk on Mueller–Hinton agar plate inoculated with the test organism; the presence of a keyhole effect was recorded 24 h after incubation. Resistance to carbapenems was detected as described by Birgy et al. (14). In this method, ertapenem, imipenem, and meropenem were tested on Mueller–Hinton agar plates (MHA) impregnated with 5 mM of EDTA, 10 mg/mL of phenyl boronic acid (PBA), and 250 μg/mL cloxacillin (embedded) were used, respectively, for the detection of metallo-β-lactamases (MBLs), *Klebsiella pneumoniae* carbapenemases (KPCs), and overproduction of AmpC. Additionally, disks of amoxicillin–clavulanic acid, ceftazidime, cefotaxime, and cefepime were added and arranged in a manner where a keyhole effect could be observed for the detection of ESBL production, and this provided an additional step for ESBL detection in view of the enhanced keyhole effect observed in presence of EDTA and/or PBA. Resistance to temocillin was used for the phenotypic detection of Oxa-48 carbapenemase. *E. coli* ATCC 25922 was used as ESBL-negative and *K. pneumoniae* 700603 was used as ESBL-positive reference strains.

### Genotypic detection of resistance genes

Multiplex PCR (four plex) was used to determine the presence bla_TEM_, bla_SHV_, bla_CTX-M_, and bla_OXA_ (15). Primers used for these genes were: blaSHV – 237bp (F5′-CTTTATCGGCCCTCA CTCAA-3′, R5′-AGGTGCTCATCATGGGAAAG-3′), blaTEM – 445bp (F5′-CGCCGCATACACTATTCTCAGAATGA-3 ′, R5′-ACGCTCACCGGCTCCAGATTTAT-3′), blaCTX-M – 593 bp (5′-ATGTGCAGYACCAGTAARGTKATGGC-3′, R5′-TGGGTR AARTARGTSACCAGAAYCAGCGG-3′), blaOXA – 813 bp (F5′-ACACAATACATATCAACTTCGC-3′, R5′-AGTGTGTTTAGA ATGGTGATC-3′). PCR was performed as follows: 15 min at 95°C, 30 s at 94°C (30 cycles), 90 s at 62°C (30 cycles), 60 s at 72°C (30 cycles), 10 min at 72°C. The amplicons were tested by gel electrophoresis. The PCR mix of this reaction included 10 μl of master mix, 4 μl of primers mix, 1 μl of DNA, 5 μl of water.

For the detection of carbapenemases, 2 μl of total DNA was subjected to multiplex PCR in a 50-μL reaction mixture. The mix for the detection of bla_IMP_, bla_VIM_, and bla_SPM_ genes contained 1× PCR buffer [10 mmol/L Tris–HCl (pH 8.3), 50 mmol/L KCl], 1.5 mmol/L of MgCl_2_, 0.125 mmol/L of each deoxynucleotide triphosphate, 10 μmol/L of each primer, and 2 U of AmpliTaq Gold Polymerase (Roche, Meylan, France). The mix for the detection of bla_KPC_, bla_NDM_, bla_BIC_, and bla_OXA-48_ was the same concentration. The mix for the detection of bla_AIM_, bla_DIM_, bla_GIM_, and bla_SIM_ contained 1× PCR buffer [10 mmol/L Tris–HCl (pH 8.3), 50 mmol/L KCl], 3 mmol/L of MgCl_2_, 0.125 mmol/L of each deoxynucleotide triphosphate, 10 μmol/L of each primer, 3 μL of dimethyl sulfoxide, and 2 U of AmpliTaq Gold Polymerase. Amplification was carried out with the following thermal cycling conditions: 10 min at 94°C and 36 cycles of amplification consisting of 30 s at 94°C, 40 s at 52°C, and 50 s at 72°C, with 5 min at 72°C for the final extension. DNA fragments were analyzed by electrophoresis in a 2% agarose gel at 100 V for 1 h in 1× TAE [40 mmol/L Tris–HCl (pH 8.3), 2 mmol/L acetate, 1 mmol/L EDTA] containing 0.05 mg/L ethidium bromide. The primers were as follows (5’–3’): *bla*_IMP_ – 232 bp (F GGAATA GAGTGGCTTAAYTCTC, R GGTTTAAYAAAACAACCACC), *bla*_SPM_ – 271 (F AAAATCTGGGTACGCAAACG, R ACATTAT CCGCTGGAACAGG), *bla*_AIM_ – 322 (F CTGAAGGTGTACGGAAACAC, R GTTCGGCCACCTCGAATTG), *bla*_VIM_ – 390 (F GATGGTGTTTGGTCGCATA, R CGAATGCGCAGCACCAG), *bla*_OXA-48_ – 438 (F GCGTGGTTAAGGATGAACAC, R CATC AAGTTCAACCCAACCG), *bla*_GIM_ – 477 (F TCGACACACCTTGGTCTGAA, R AACTTCCAACTTTGCCATGC), *bla*_BIC_ – 537 (FTATGCAGCTCCTTTAAGGGC, R TCATTGGCGGTGC CGTACAC), *bla*_SIM_ – 570 (F TACAAGGGATTCGGCATCG, R TAATGGCCTGTTCCCATGTG), *bla*_NDM_ – 621 (F GGTTTGGCGATCTGGTTTTC, R CGGAATGGCTCATCACGATC), *bla*_DIM_ – 699 (F GCTTGTCTTCGCTTGCTAACG, R CGTT CGGCTGGATTGATTTG), *bla*_KPC_ – 798 (F CGTCTAGTTCTGCTGTCTTG, R CTTGTCATCCTTGTTAGGCG).

### Genotyping

Pulsed-field gel electrophoresis (PFGE) was carried out using restriction enzyme *Xba*I (Invitrogen) and the PFGE CHEF MAPPER (Bio-Rad). The comparison for *E. coli* was made using the Dice coefficient. The dendrograms were generated adopting the UPGMA method (unweighted pair group method using arithmetic averages) using Bionumerics software. DNA ratio was calculated on the Dice coefficient, and isolates were considered genetically related if the Dice coefficient correlation was 85% or higher. Isolates with 85% and above relatedness were clustered using a red frame.

## Results

**Figure 1:**
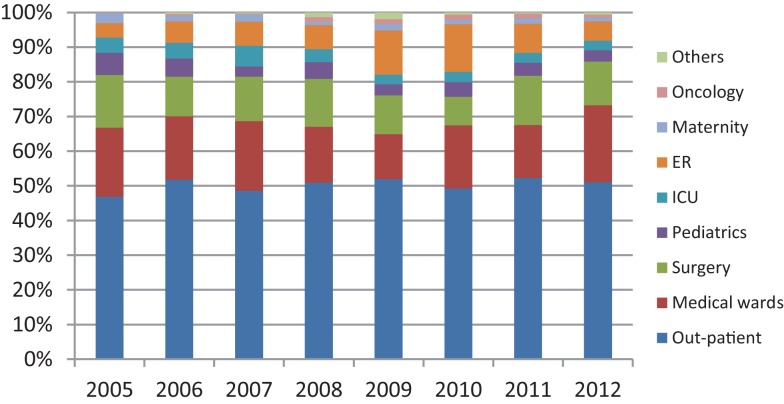
**Distribution of urinary *E. coli* in percent by location and year**.

Over the 8-year period, a total of 6284 Gram-negative bacterial isolates was recovered from documented UTIs. Table 1 shows the Gram-negative bacilli most frequently isolated from these positive urine cultures. As expected, the highest frequency was observed with *E. coli* (60.53–73.98%) followed by *K. pneumoniae* (5.32–8.33%), *Proteus mirabilis* (3.36–6.17%), *Pseudomonas aeruginosa* (2.3–3.9%), and *Enterobacter cloacae* (0.76–2.23%). Figure 1 shows the distribution of urinary isolates of *E. coli* by location. Out-patient isolates predominate over the years with a more or less stable percent of occurrence.

**Table 1:** **Frequency of Gram-negative Bacilli-documented UTI isolates at the CHN hospital classified by year**.

	Total Nb of isolates	Bacteria most frequently isolated from urinary tract infections									
		*Escherichia coli*		*Klebsiella pneumoniae*		*Enterobacter cloacae*		*Proteus mirabilis*		*Pseudomonas aeruginosa*	
2005	615	412	66.99%	38	6.18%	8	1.30%	25	4.07%	19	3.09%
2006	564	409	72.52%	30	5.32%	7	1.24%	24	4.26%	22	3.90%
2007	525	358	68.19%	29	5.52%	4	0.76%	30	5.71%	20	3.81%
2008	684	506	73.98%	57	8.33%	12	1.75%	23	3.36%	24	3.51%
2009	718	516	71.87%	56	7.80%	16	2.23%	41	5.71%	15	2.09%
2010	843	600	71.17%	61	7.24%	10	1.19%	52	6.17%	21	2.49%
2011	1162	694	59.72%	87	7.49%	16	1.38%	58	4.99%	38	3.27%
2012	1173	710	60.53%	91	7.76%	13	1.11%	59	5.03%	27	2.30%

**Figure 2:**
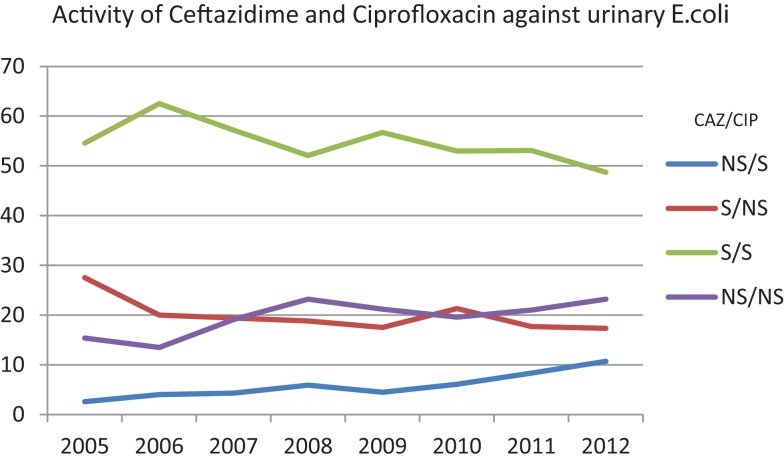
**Percentages of susceptibility of urinary *E. coli* to ceftazidime and ciprofloxacin over the years**. NS/S: trends of the isolates non-susceptible to ceftazidime/susceptible to ciprofloxacin. S/NS: trends of the isolates susceptible to ceftazidime/non-susceptible to ciprofloxacin. S/S: trends of the isolates susceptible to ceftazidime/susceptible to ciprofloxacin. NS/NS: trends of the isolates non-susceptible to ceftazidime/non-susceptible to ciprofloxacin.

Figure 2 is plotted based on scatterplot analysis of co-susceptibility/non-susceptibility to ceftazidime and ciprofloxacin. Over the years, the isolation of *E. coli* that are susceptible to both antibiotics is decreasing (62.5% in 2006 and 48.7% in 2012), while strains that are non-sensitive to both antibiotics are increasing (13.5% in 2006 and 23.2% in 2012).

**Figure 3:**
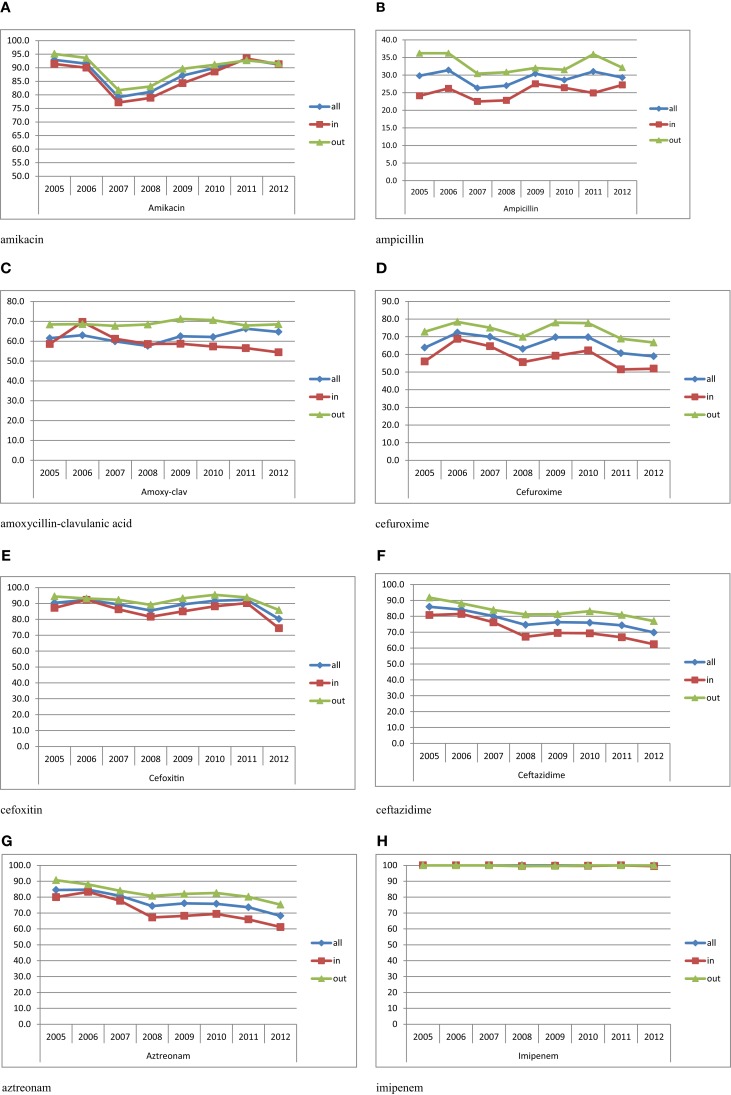

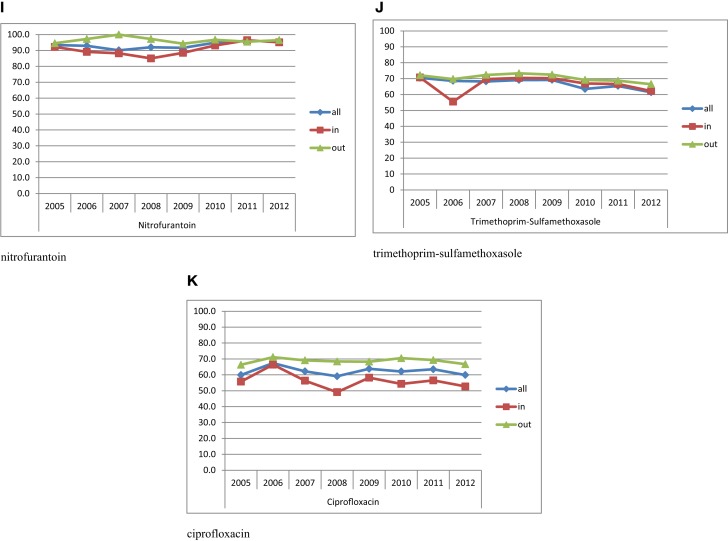
**Percentages of susceptibility of urinary isolates of *E. coli* in all/in/and out-patients**. **(A)** Amikacin. **(B)** Ampicillin. **(C)** Amoxicillin–clavulanic acid. **(D)** Cefuroxime. **(E)** Cefoxitin. **(F)** Ceftazidime. **(G)** Aztreonam. **(H)** Imipenem. **(I)** Nitrofurantoin. **(J)** Trimethoprim–sulfamethoxazole. **(K)** Ciprofloxacin.

Table 2 shows the susceptibility profiles of *E. coli* to the different antimicrobial agents. No major difference in susceptibility is observed between the overall isolates and the urinary isolates. The lowest percentage of susceptibility was manifested against ampicillin reaching 26% in 2007. Over successive years, the susceptibility to third and fourth generations’ cephalosporins (represented by Ceftazidime in Figure 3F) and aztreonam (Figure 3G) shows an obvious and constant decrease; this is coupled by an increase in the production of ESBL as shown in Table 2 (12.6% in 2005 and 29.4% in 2012 for all *E. coli* isolates). A similar pattern of ESBL production is noted for the urinary isolates (11.6% in 2005 and 25.3% in 2012). Since 2008, a minimum of 10% susceptibility decrease to third and fourth generation cephalosporin between in and out-patients isolates was observed. Such a difference is most likely due to the higher occurrence of ESBL and AmpC in the hospital isolates. Figure 3K shows a considerably higher susceptibility of isolates from outpatients to ciprofloxacin, although the general patterns of in- and out-patients’ isolates are similar. The susceptibility to trimethoprim–sulfamethoxazole is essentially the same for in and outpatients; however, it is decreasing with time (Figure 3J). Susceptibility to nitrofurantoin manifests an irregular pattern where the high rates in 2007 are significantly decreased in in-patients, affecting therefore the overall percentage of susceptibility; however, this antibiotic seems to keep its high activity against *E. coli*. Two imipenem-resistant *E. coli* were isolated from UTIs in 2011 and 2012; of course, this does not affect the pattern of susceptibility to this antibiotic (Figure 3H). Isolates with decreased susceptibility to fosfomycin were observed in 2011 and 2012. Figures 3A and 3E show the patterns of susceptibility to amikacin and cefoxitin respectively.

**Table 2:** **The rates of susceptibility of urinary and all isolates of *E. coli* between 2005 and 2012 is shown**.

	Percentages of susceptibility of *E. coli* isolated between 2005 and 2012															
	2005		2006		2007		2008		2009		2010		2011		2012	
	Urine (412)	All (454)	Urine (408)	All (493)	Urine (358)	All (484)	Urine (506)	All (629)	Urine (516)	All (650)	Urine (600)	All (743)	Urine (694)	All (821)	Urine (710)	All (831)
Amikacin	92.9	92.0	91.5	93.0	79.2	81.0	81.1	81.0	87.1	88.0	89.9	90.0	93.0	94.0	91.2	93.0
Amoxi–Clav	59.0	61.5	63.2	63.0	55.7	59.9	58.8	57.6	61.4	62.5	63.4	62.1	65.5	66.3	63.5	64.7
Ampicillin	29.8	29.0	31.4	30.0	26.3	26.0	27.0	26.0	30.4	29.0	28.6	28.0	31.0	34.0	29.3	31.0
Aztreonam	84.5	83.0	84.7	85.0	80.8	79.0	74.4	74.0	76.1	76.0	75.8	75.0	73.6	76.0	68.2	71.0
Cefepime	86.2	85.0	86.3	86.0	80.1	79.0	77.1	77.0	77.1	77.0	76.6	76.0	76.4	79.0	71.4	74.0
Cefotaxime	86.3	85.0	84.4	84.0	81.1	79.0	73.8	73.0	75.7	75.0	74.2	74.0	72.7	75.0	67.6	71.0
Cefoxitin	90.4	89.0	92.2	94.0	89.4	88.0	85.5	87.0	89.4	90.0	91.7	91.0	92.3	92.0	80.2	82.0
Ceftazidime	86.0	85.0	84.2	84.0	80.1	79.0	74.6	74.0	76.3	76.0	76.0	76.0	74.3	77.0	69.8	73.0
Cefuroxime	63.8	63.0	72.3	74.0	69.9	69.0	63.1	63.0	69.7	69.0	69.7	69.0	60.7	64.0	58.9	62.0
Ciprofloxacin	59.9	60.0	67.4	69.0	62.2	63.0	59.1	60.0	63.8	62.0	62.1	62.0	63.5	68.0	59.9	66.0
Fosfomycin	100.0	100.0	100.0	100.0	100.0	100.0	100.0	100.0	100.0	100.0	100.0	100.0	99.4	99.5	99.6	99.6
Gentamicin	61.8	70.0	59.6	68.0	53.6	50.0	54.8	51.0	56.5	58.0	51.4	51.0	52.4	46.0	54.3	55.0
Imipenem	100.0	100.0	100.0	100.0	100.0	100.0	100.0	100.0	100.0	100.0	100.0	100.0	99.9	99.8	99.9	99.6
Nitrofurantoin	93.4	94.0	92.9	93.0	90.1	91.0	92.0	92.0	91.6	92.0	94.9	95.0	95.9	97.0	95.9	96.0
Piperacillin	32.1	32.0	36.4	34.0	29.9	30.0	34.1	33.0	36.6	35.0	32.6	34.0	33.4	39.0	32.6	34.0
Pip-Tazo	86.3	85.0	86.4	86.0	71.2	70.0	74.5	75.0	79.5	80.0	85.5	87.0	87.4	89.0	84.1	84.0
Tigecycline	ND	ND	ND	ND	ND	ND	ND	ND	ND	ND	ND	ND	ND	100.0	100.0	100.0
Tobramycin	67.8	67.0	70.0	70.0	56.6	56.0	51.4	53.0	59.0	59.0	48.8	48.0	42.4	47.0	49.2	53.0
Co-trimoxasole	68.6	70.0	68.1	68.0	67.4	70.0	69.4	70.0	68.7	69.0	65.5	64.0	63.3	65.0	58.4	62.0

ESBL rate (%)	11.6	12.6	13.9	13.9	17.3	18.3	21.8	23.6	21.1	22.1	21.9	23.4	23.8	24.6	25.3	29.4

*ESBL, extended-spectrum beta lactamase*.

Tables 3 and 4 report the susceptibility profiles of the urinary isolates of *E. coli* by location. ICU isolates were constantly associated with the lowest rates of susceptibility to extended-spectrum cephalosporins, ciprofloxacin, as well as most of the tested antibiotics. Isolates coming from ER patients showed rates of susceptibility similar to out-patients’ isolates.

**Table 3:** **Profiles of susceptibility of urinary *E. coli* isolated between 2005 and 2008 grouped by location**.

Percentages of susceptibility of urinary isolates of *E. coli* 2005–2008														
	Nb (%)	AMK	AMP	AUG	CFX	FOX	CTX	CAZ	FEP	AZT	IMP	CIP	NIT	SXT
**2005**														
Urinary samples from all patients (412)		92.9	29.8	43.2	63.8	90.4	86.3	86.0	86.2	84.5	100	59.9	93.4	70.5
Out-patient	193 (46.81)	95.1	36.2	48.8	72.8	94.4	91	91.8	92.3	90.7	100	66.3	94.5	72.1
Medical wards	83 (20)	90.8	24.2	41.8	52.7	83.9	80.3	76.9	79.3	75.9	100	50.6	90	70.1
Surgery	63 (15.16)	92.1	24.6	30.4	52.2	85.7	81.2	79.4	79.4	79.4	100	49.2	90.9	71.6
Pediatrics	26 (6.37)	83.3	20.7	37.9	75.9	100	84.2	85.7	83.3	83.3	100	87.5	100	71.1
ICU	18 (4.4)	95	15	20	35	80	68.8	75	75	75	100	35	88.9	68.6
ER	17 (4.18)	94.1	26.3	47.4	57.9	94.1	93.3	89.5	88.2	88.2	100	70.6	94.4	71.5
Maternity	12 (2.86)	100	38.5	69.2	84.6	91.7	100	100	100	100	100	66.7	100	71.5
**2006**														
Urinary samples from all patients (408)		91.5	31.4	53.2	72.3	92.2	84.4	84.2	86.3	84.7	100	67.4	92.9	68.6
Out-patient	211 (51.78)	93.6	36.2	61.5	78.4	93.1	87.6	88.1	90.3	88	100	71.1	97.2	69.7
Medical wards	75 (18.29)	89.6	20.8	35.1	61	84.4	76.6	72.7	76.6	76.6	100	57.1	83.1	68.1
Surgery	48 (11.4)	93.8	41.7	64.6	81.2	93.8	93.8	97.9	95.8	95.8	100	62.5	87.5	7.2
ER	26 (6.18)	92.3	23.1	53.8	84.6	100	92.3	88.5	92.3	92.3	100	80.8	96.2	68.9
Pediatrics	23 (5.23)	95.5	18.2	27.3	50	100	68.2	63.6	68.2	63.6	100	86.4	90.9	67.4
ICU	18 (4.51)	63.2	21.1	36.8	36.8	84.2	68.4	73.7	73.7	73.7	100	31.6	89.5	69.7
Maternity	7 (1.66)	100	42.9	42.9	85.7	100	85.7	85.7	85.7	85.7	100	100	100	69.2
**2007**														
Urinary samples from all patients (358)		79.2	26.3	40.7	69.9	89.4	81.1	80.1	80.1	80.8	100	62.2	90.1	68.2
Out-patient	174 (48.53)	81.7	30.4	44.2	75.1	92.3	85	84	84.5	84	100	69.1	100	72.3
Medical wards	72 (20.11)	82.7	29.3	40	61.3	89.3	80	80	74.7	80	100	61.3	80.8	69.7
Surgery	46 (12.87)	79.2	22.9	37.5	70.8	85.4	77.1	75	79.2	78.7	100	47.9	90.9	70.8
ER	25 (6.97)	84.6	23.1	38.5	76.9	80.8	88.5	84.6	88.5	88.5	100	61.5	100	69.4
ICU	22 (5.9)	45.5	4.5	18.2	31.8	72.7	45.5	45.5	45.5	45.5	100	27.3	85.3	67.8
Pediatrics	11 (2.95)	81.8	18.2	72.7	81.8	100	81.8	81.8	81.8	81.8	100	90.9	100	70.5
Maternity	8 (2.14)	75	12.5	12.5	87.5	100	100	100	100	100	100	75	94.4	68.9
**2008**														
Urinary samples from all patients (506)		81.1	27.0	48.8	63.1	85.5	73.8	74.6	77.1	74.4	100	59.1	92.0	69.1
Out-patient	264 (50.96)	83.1	30.8	53.8	69.9	89.1	81.6	81.2	83.8	80.8	99.6	68.4	97.2	73.3
Medical wards	83 (16.09)	79.8	20.2	39.3	48.8	76.2	59.5	58.3	64.3	61.9	100	40.5	90	69.8
Surgery	71 (13.79)	81.9	23.6	45.8	55.6	77.8	66.7	68.1	70.8	68.1	100	43.1	100	70.6
ER	36 (6.9)	83.3	36.1	58.3	72.2	94.4	80.6	88.9	86.1	83.3	100	80.6	91.7	72.1
Pediatrics	25 (4.79)	72	24	44	64	100	68	76	76	72	96	68	42.9	71.3
ICU	20 (3.83)	65	15	30	45	75	45	45	45	45	100	35	50	68.4
Oncology	7 (1.34)	78.55	8.35	39.3	55.9	70.2	77.35	77.35	77.35	77.35	100	29.8	97.15	70.4

**Table 4:** **Profiles of susceptibility of urinary *E. coli* isolated between 2009 and 2012 grouped by location**.

Profiles of susceptibility of urinary isolates of *E. coli* 2009–2012														
	Nb (%)	AMK	AMP	AUG	CFX	FOX	CTX	CAZ	FEP	AZT	IMP	CIP	NIT	SXT
**2009**														
Urinary samples from all patients (516)		87.1	30.4	41.4	69.7	89.4	75.7	76.3	77.1	76.1	100	63.8	91.6	69.2
Out-patient	268 (51.87)	89.6	32	44.6	78	93.2	81.3	81.3	83.1	82	99.6	68.3	94.2	72.5
Medical wards	109 (13.06)	80	21.4	27.1	45.7	81.4	55.7	57.1	58.6	54.3	100	51.4	84.3	69.3
ER	85 (12.69)	92.6	42.6	51.5	83.8	94.1	89.7	91.2	89.7	91.2	100	75	91.2	71.9
Surgery	49 (11.19)	78.3	21.7	35	56.7	76.7	65	66.7	65	65	98.3	53.3	86.7	71.1
Pediatrics	25 (3.17)	94.1	23.5	23.5	64.7	94.1	64.7	64.7	64.7	64.7	100	70.6	100	67.4
ICU	18 (2.8)	66.7	6.7	13.3	13.3	60	40	40	40	40	100	6.7	93.3	70.1
Maternity	10 (1.87)	90	30	60	70	100	90	90	90	90	100	70	80	70.1
Oncology	7 (1.4)	94.45	44.45	61.15	72.25	100	80.55	80.55	80.55	80.55	100	63.9	91.65	71.1
**2010**														
Urinary samples from all patients (600)		89.9	28.6	47.8	69.7	91.7	74.2	76.0	76.6	75.8	100	62.1	94.9	63.5
Out-patient	295 (49.19)	91.1	31.5	51.1	77.7	95.4	82	83.2	83.6	82.6	100	70.5	96.7	69.2
Medical wards	109 (18.23)	91.2	16.8	33.6	50.4	84.1	57.5	59.6	61.9	61.1	100	43.4	88.5	64.2
ER	99 (13.71)	88.2	34.1	56.5	76.5	97.6	80	78.8	80	80	100	70.6	95.3	68.9
Surgery	59 (8.23)	86.3	31.4	49	64.7	86.3	68.6	71.4	70.6	68.6	100	39.2	94.1	68.6
Pediatrics	26 (4.19)	92.3	26.9	61.5	69.2	84.6	69.2	73.1	73.1	73.1	100	69.2	96.2	68.1
ICU	20 (3.06)	73.7	0	0	26.3	68.4	31.6	52.6	42.1	47.4	100	31.6	100	63.4
Oncology	11 (1.61)	90	50	50	70	100	70	66.7	80	70	100	70	90	69.1
Maternity	8 (1.13)	100	57.1	71.4	85.7	85.7	100	100	100	100	100	100	100	68.7
**2011**														
Urinary samples from all patients (694)		93.0	31.0	67.0	60.7	92.3	72.7	74.3	76.4	73.6	99.9	63.5	95.9	65.4
Out-patient	363 (52.27)	92.7	35.9	73.3	68.9	93.8	79.2	80.9	81.8	80.2	100	69.3	95.5	68.7
Medical wards	105 (15.21)	93.5	22	54.5	45.5	86.9	56.1	56.1	61.8	56.1	99.0	48.8	95.1	65.4
Surgery	99 (14.23)	93.9	29.8	67.5	50.9	92	62.3	66.7	70.2	64.9	100	57.9	95.6	68.6
ER	59 (8.47)	94.1	30.9	67.6	63.2	100	79.4	79.4	79.4	79.4	100	55.9	98.5	66.9
Pediatrics	26 (3.8)	90.3	19.4	58.1	54.8	83.9	71	74.2	77.4	71	100	74.2	100	64.1
ICU	20 (2.82)	90.9	13.6	31.8	36.4	77.3	59.1	59.1	63.6	59.1	100	54.5	95.5	64.3
Maternity	11 (1.47)	91.7	16.7	58.3	83.3	83.3	100	100	100	100	100	83.3	100	67.7
Oncology	8 (1.35)	100	14.3	41.65	35.3	100	63.7	63.7	63.7	63.7	100	54.15	93.75	67.4
**2012**														
Urinary samples from all patients (710)		91.2	29.3	62.2	58.9	80.2	67.6	69.8	71.4	68.2	99.9	59.9	95.9	61.4
Out-patient	362 (51.01)	91.6	32.1	68.1	66.7	85.8	75.3	77	78.8	75.3	100	66.7	96.5	66.5
Medical wards	158 (22.25)	87.7	26.7	51.3	49.7	68.4	58.3	58.8	60.4	59.4	99.4	47.1	94.5	61.7
Surgery	89 (12.54)	93.4	25.5	60.4	51.9	75.5	61.3	64.8	66	62.3	100	49.1	94.3	62.5
ER	40 (5.56)	95.7	42.6	80.9	72.3	91.5	83	85.1	87.2	85.1	100	68.1	100	65.5
Pediatrics	23 (3.31)	96.4	25	60.7	57.1	82.1	60.7	64.3	64.3	60.7	100	75	89.3	60.6
ICU	19 (2.72)	95.7	8.7	30.4	21.7	73.9	26.1	30.4	39.1	21.7	95.7	56.5	100	60.3

A total of 88 isolates isolated in 2012 were subjected to molecular testing for ESBL enzymes. Our results suggest a high correlation between phenotypic and genotypic testing. A 100% occurrence of CTX-M in ESBL-producing isolates is recorded (Table 5), followed by TEM, SHV, and OXA. In addition, 15.9% harbored 4 different ESBL enzymes and only 13 isolates (14.8%) harbored only 1 enzyme (CTX-M).

**Table 5:** **Phenotypic and genotypic detection of the mechanisms of resistance in 88 urinary *E. coli* isolated in 2012**.

Antimicrobial susceptibility patterns and genotypic profiles of ESBL-producing urinary isolates of *Escherichia coli*												
		Phenotype	Genotype of resistance				CTX-M + SHV + TEM + OXA	CTX-M + SHV + TEM	CTX-M + TEM	CTX-M + OXA	CTX-M + SHV	CTX-M
Male	Female	ESBL	CTX-M	SHV	OXA	TEM	
29	59	(AMC-G3C)	88	27	26	60	14	11	35	12	2	13
(32.96%)	(67.04%)	88 (100%)	(100%)	(30.69%)	(29.55)	(68.19%)	(15.9%)	(12.5%)	(39.7%)	(16.6%)	(2.3%)	(14.8%)

On the other hand, concerning the five carbapenem-resistant isolates of *E. coli* (two were urinary isolates), both phenotypic and genotypic testing showed that OXA-48 was the most common mechanism of carbapenem resistance (Table 6). Two of these isolates came from pus, two from urine, and one from sputum cultures. In both urinary isolates, OXA-48 was responsible for resistance to imipenem.

**Table 6:** **Phenotypic and genotypic characterization of the mechanisms of resistance in 5 *E. coli* isolated in 2012**.

Antimicrobial susceptibility patterns and genotypic profiles of Carbapenems non-susceptible *Escherichia coli* isolates													
Suggested phenotype of resistance						Genotype of resistance							
Specimen	ESBL (AMC-G3C)	MBL (EDTA)	KPC-type	AmpC	OXA-48 (TMC)	ESBL	IMP	NDM-1	VIM	OXA-48	SIM	SPM	KPC
			(PBA and CLOXA)									
Pus	+	+	−	−	−	CTX-M	−	+	−	−	−	−	−
						TEM	
Urine	−	−	−	−	+	−	−	−	−	+	−	−	−
Sputum	+	−	−	−	+	CTX-M	−	−	−	+	−	−	−
						SHV	
Pus	+	−	−	−	+	CTX-M	−	−	−	+	−	−	−
						TEM	
Urine	+	−	−	−	+	CTX-M	−	−	−	+	−	−	−
						TEM	

*ESBL, extended-spectrum beta-lactamase; MBL, metallo-beta lactamase; KPC, karbapenemase producing *klebsiella;* AMC, amoxicillin–clavulanic acid; G3C, third generation cephalosporin, TMC, temocillin*.

**Figure 4:**
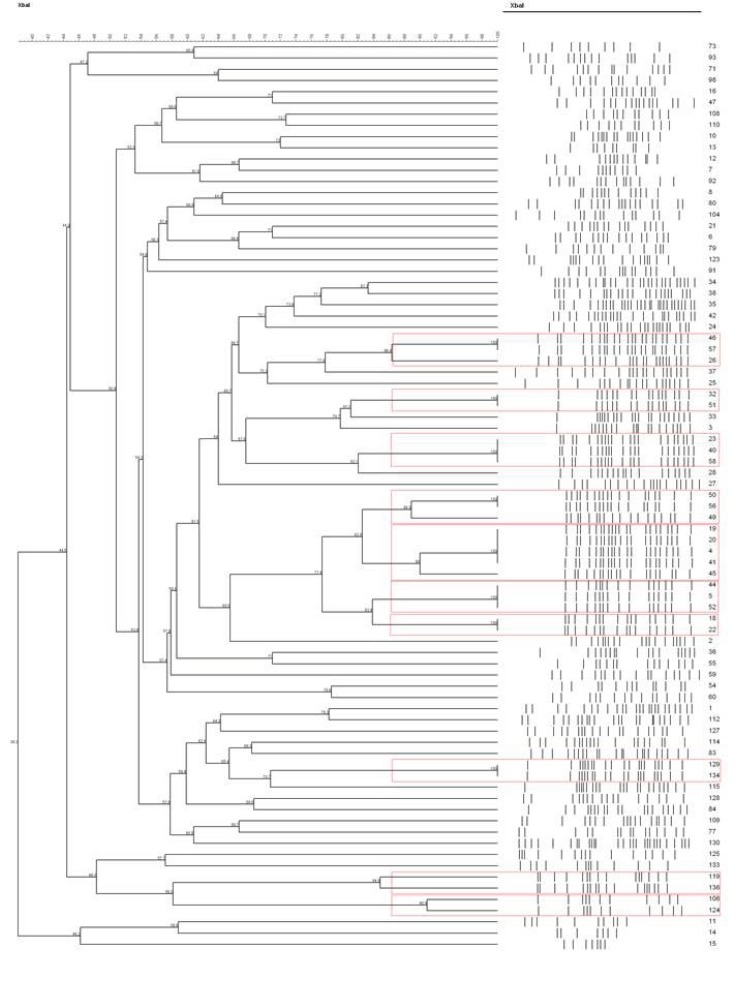
**Dendrogram generated by the PFGE of 88 urinary isolates of *E. coli* (2012)**.

On the other hand, the PFGE results of *E. coli* isolated in 2013 (Figure 4) demonstrated that 10 clusters were generated, denoting diversity among detected isolates. However, those isolates falling within the same cluster have a relatively high degree of relatedness among them.

## Discussion

Lebanon is currently witnessing an increase in the number of bacterial infections that are associated with a wide spectrum of resistance to common antimicrobial agents (16). The presence of ESBL-producing organisms has been reported in hospitals as well as in the community (17). In 1997, the percentage of ESBL-producing *E. coli and K. pneumoniae* was 1.3 and 7.5%, respectively, as compared to non-ESBL-producing strains of the same species. This percentage, however, has increased over the years [in 2001, the percentage of ESBL-producing *E. coli* reached 4% (16, 18)]. Overall, there is a scarcity of data relating to antimicrobial resistance in the Middle East (16). Molecular analysis revealed that CTX-M-15 is the most widespread ESBL since it was present in 83% of the resistant strains. The reason behind this increase of the frequency of occurrence of ESBL-producing organisms is likely due to the misuse, and abuse of antibiotics (19).

Our results show that the northern part of Lebanon is not different from the rest of the country. The rate of ESBLs increased considerably over the course of the study period from 12.6 to 29.4 in all *E. coli* isolates and from 11.6 to 25.3% in urinary isolates. This result correlates with the general trend in the country. In addition to containing CTX-M-15, 30.7% of isolates tested positive for carriage of SHV, 29.6% for OXA, and 68.2% for TEM. These results indicate that ESBL-producing organisms frequently harbor more than one β-lactamase gene. In fact, 15.9% of *E. coli* harbored all four ESBL enzymes and 14.8% harbored only one gene of resistance (Table 5). Though this does not impact the treatment strategy, harboring more than one gene of ESBL enzymes makes the diversity of resistance gene carriage considerably bigger.

It is known that the occurrence of ESBL-producing *E. coli* in high-risk areas of the hospital, such as ICUs, has increased significantly (20, 21). In this context, bigger concern has resulted from reports of the increasing frequency of ESBL-producing organisms causing infections in outpatients. In our study, most UTIs were isolated from out-patients. This suggests an important spread of these enzymes in the community, even if these outpatients’ infections are not entirely community acquired and might correlate with hospital or health care facilities visits.

Appropriate antibiotic treatment for infections caused by ESBL-producing *E. coli* is significantly affected by the cross-resistance to other antibiotics such as quinolones (22). Resistance to these drugs has been shown to be related to the presence of a conjugative plasmid associated with the ESBL phenotype. In this context, carbapenems represent a good choice when therapy is needed for serious infections.

In our study, co-resistance has been observed with fluoroquinolones and ceftazidime (Figure 2). While susceptible isolates to both antibiotics remain the majority, the trend of occurrence of these isolates is clearly decreasing over the years and is coupled by an increase in resistance to either antibiotics. This implies that fluoroquinolone resistance could be driven by cephalosporin use at both hospital and community levels. Another implication of this observation is that ciprofloxacin resistance may be associated with limited treatment options for other classes of agents, as observed in our study. The isolation of two carbapenem-resistant *E. coli* from UTIs should be carefully considered; their occurrence foreshadows of a possible spread of carbapenemases into the community.

Although the PFGE results show 10 clusters among the urinary isolates of *E. coli* collected in 2013 with high level of similarity (>95%), these results indicate genetic diversity yet all encoded the CTX-M-15 type enzyme.

Limitations of the study include that some of the isolates included in the analysis may have been associated with asymptomatic bacteriuria rather than true UTI. While we used standard surveillance criteria to define UTI, we allowed for some isolates to be included without meeting those criteria if their clinical picture was highly consistent with UTI. This had the potential to introduce inter-observer variability in the determination of which isolates were included. Furthermore, asymptomatic bacteriuria is frequently associated with both high colony counts of bacteria and significant pyuria, especially in the presence of a bladder catheter. While the epidemiology of isolates obtained from patients with asymptomatic bacteriuria should be similar to those from patients with true infection, the public health implications are different, since asymptomatic bacteriuria does not require treatment except in rare circumstances.

In conclusion, ESBL-producing *E. coli* in urinary isolates is a growing problem and is spreading over the whole country. This study is among the first to provide information on the prevalence and distribution of isolated *E. coli* with ESBL producer, possible types of enzymes produced, clonal relationship, and susceptibility to potentially active drugs in the north (2005–2012). This information contributes to the understanding of the epidemiology of resistance in the whole country as well as the implementation of recommendations for the management of antimicrobials, infection control measures, and active surveillance and antimicrobial stewardship is highly needed.

## Conflict of Interest Statement

The authors declare that the research was conducted in the absence of any commercial or financial relationships that could be construed as a potential conflict of interest.
