# Corrigendum: *Escherichia coli* Isolated from Urinary Tract Infections of Lebanese Patients between 2005 and 2012: Epidemiology and Profiles of Resistance

**DOI:** 10.3389/fmed.2015.00066

**Published:** 2015-09-22

**Authors:** Ziad Daoud, Elie Salem Sokhn, Khalil Masri, Katia Cheaito, Nathaline Haidar-Ahmad, Ghassan M. Matar, Shira Doron

**Affiliations:** ^1^Faculty of Medicine and Medical Sciences, University of Balamand, Tripoli, Lebanon; ^2^“Centre Hospitalier du Nord” Hospital, Zgharta, Lebanon; ^3^Faculty of Medicine, American University of Beirut, Beirut, Lebanon; ^4^Division of Geographic and Infectious Diseases, Tufts Medical Center, Boston, MA, USA

**Keywords:** ESBL, carbapenemases, *E. coli*, urinary infections, bacterial resistance

Inadvertently, the names of two authors were not included in the final version of the manuscript sent for typesetting and this oversight was not picked up during the final proofs. The authors regret the oversight and any inconvenience these may have caused.

The two missing authors are:

Katia Cheaito

Nathaline Haidar-Ahmad

The final author contributions are as follows:

Ziad Daoud participated in designing the study and in the lab work, as well as in writing all the sections of the article.

Elie Salem Sokhn participated in designing the study and in the lab work, as well as in writing all the sections of the article.

Khalil Masri participated in designing the study and in the clinical part of the work, as well as in writing all the sections of the article.

Katia Cheaito participated in designing the study and in the lab work (mainly the PFGE), as well as in writing all the sections of the article.

Nathaline Haidar-Ahmad participated in designing the study and in the lab work (mainly the PFGE), as well as in writing all the sections of the article.

Ghassan Matar participated in designing the study and in the lab work (mainly the PFGE), as well as in writing all the sections of the article.

Shira Doron participated in designing the study and in clinical protocols elaboration, as well as in writing and reviewing all the sections of the article.

The original article has been updated.

## Conflict of Interest Statement

The authors declare that the research was conducted in the absence of any commercial or financial relationships that could be construed as a potential conflict of interest.

